# Supplementary material for: The interactome of intact mitochondria by cross-linking mass spectrometry provides evidence for coexisting respiratory supercomplexes
Source: Mol Cell Proteomics. 2017 Dec 8;17(2):216–32. doi: 10.1074/mcp.RA117.000470 (PMC5795388; doi:10.1074/mcp.RA117.000470)
Supplement: Supplemental Data [file supp_RA117.000470_133922_0_supp_23978_fzffwf.zip › spectra_annotation/mito_DR_spectra_annotation/69-1-1-1-4-1.pdf]

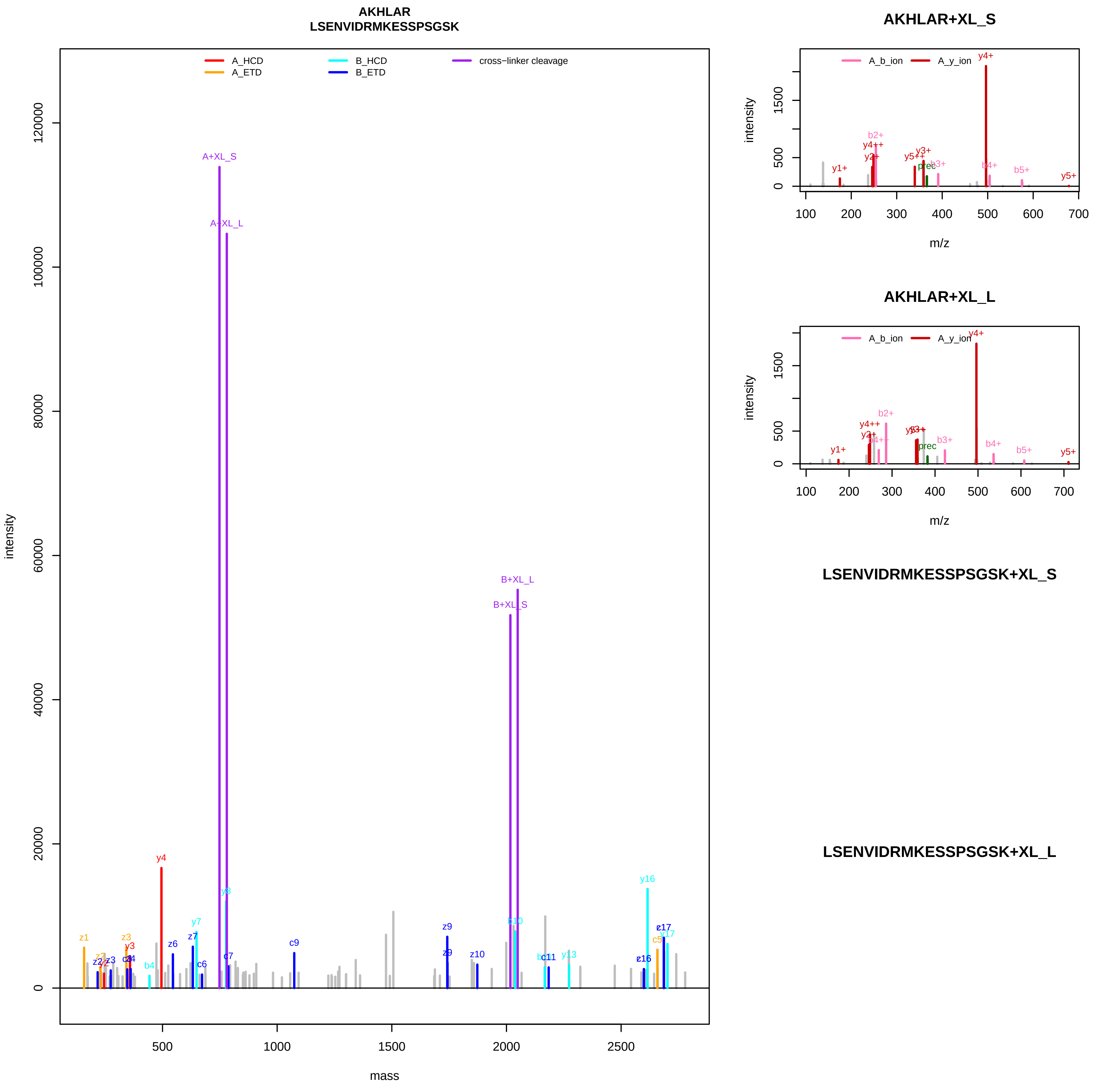
AKHLAR
LSENVIDRMKESSPSGSK
A_HCD
A_ETD
B_HCD
B_ETD
cross−linker cleavage
A+XL_S
A+XL_L
B+XL_L
B+XL_S
intensity
mass
y4
y16
y7
z9
z6
z7
c9
c7
c6
z10
z16
z17
y17
c5
c11
y13
z1
z3
y3
b4
AKHLAR+XL_S
AKHLAR+XL_L
A_b_ion
A_y_ion
m/z
y4+
b2+
y4++
y2+
y5++
y3+
prec
b3+
y1+
b4+
b5+
y5+
LSENVIDRMKESSPSGSK+XL_S
LSENVIDRMKESSPSGSK+XL_L
